# Absence of human herpes virus-8 (HHV8) in nephrogenic systemic fibrosis

**DOI:** 10.1186/1756-0500-1-82

**Published:** 2008-09-17

**Authors:** Patrick J O'Donnell, Wayne H Duke, Liron Pantanowitz

**Affiliations:** 1 Department of Pathology, Baystate Medical Center, Tufts University School of Medicine, Springfield, MA, USA

## Abstract

**Background:**

Nephrogenic systemic fibrosis (NSF) is a fibrosing disorder that exhibits CD34 expression in the majority of lesional spindle cells. Several features of NSF bear similarity to Kaposi sarcoma.

**Findings:**

Skin lesions procured from two male NSF patients were found to be negative for HHV8 (LNA-1) by means of immunohistochemsitry.

**Conclusion:**

This finding negates a role for HHV8 in the pathogenesis of NSF.

## Background

Nephrogenic systemic fibrosis (NSF) is a fibrosing disorder seen exclusively in patients with severe impairment of renal function [1]. Originally defined as a dermatologic condition called Nephrogenic Fibrosing Dermopathy (NFD), the process of NSF is now well characterized as a systemic one, with distinct pathologic changes identified throughout the body [2]. The typical dermatologic clinical presentation is characterized by symmetrical papules, nodules and brawny induration of the skin, limited to the extremities and trunk [3]. Debilitating joint contractures can also be seen.

Histopathologic changes include an increased population of mitotically inactive, bland spindle cells in the dermis which often extends into underlying subcutaneous tissue [4]. Thick collagen bundles with surrounding clefts displaying variable amounts of elastic fibers and mucin, set amongst a paucity of chronic inflammatory cells, are frequent findings [4,5]. Multinucleated cells may also be present. Immunohistochemical analysis (IHC) of tissue from NSF patients shows dual expression of CD34 and procollagen I in the majority of lesional spindle cells. These cells are postulated to represent circulating fibrocytes recruited from the bone marrow, which subsequently mediate their pathologic effects on lesional tissue [6,7].

The Centers for Disease Control and Prevention published a report in 2002 of a case controlled study of NSF patients that was unable to find any drug, toxin, or infectious agent (not specified) to explain the etiology of NSF [2]. Cowper *et al *also proved by in situ hybridization that Epstein Barr virus was not present in tissue of NSF patients [4]. However, recent evidence has shown a strong link between the development of NSF in patients with impaired renal function undergoing Magnetic Resonance (MR) studies using gadolinium based contrast media [3,8]. The exact pathogenesis between gadolinium exposure and circulating fibrocyte recruitment in NSF is currently unknown.

Several features of NSF bear similarity to Kaposi's sarcoma (KS). Lesions from both NSF and KS are comprised of CD34 positive spindled cells [9] and stroma containing procollagen type I [10], albeit KS is more vascular. Similar to NSF, the lesional cells of KS are believed to be derived from circulating CD34+ progenitor cells, which serve as reservoirs of Human Herpes Virus-8 (HHV8) [11-13]. It is well established that HHV8 infection plays an essential role in the development of all forms of KS. Moreover, both renal transplant recipients and hemodialysis patients have been shown to be at higher risk for infection with HHV8 and subsequent development of KS [14-19].

Given the aforementioned similarities, we sought to determine if HHV8 might play a role in the pathogenesis of NSF. To the best of our knowledge, there is no published data assessing for the presence of HHV8 in patients with NSF.

## Findings

**Figure 1:**
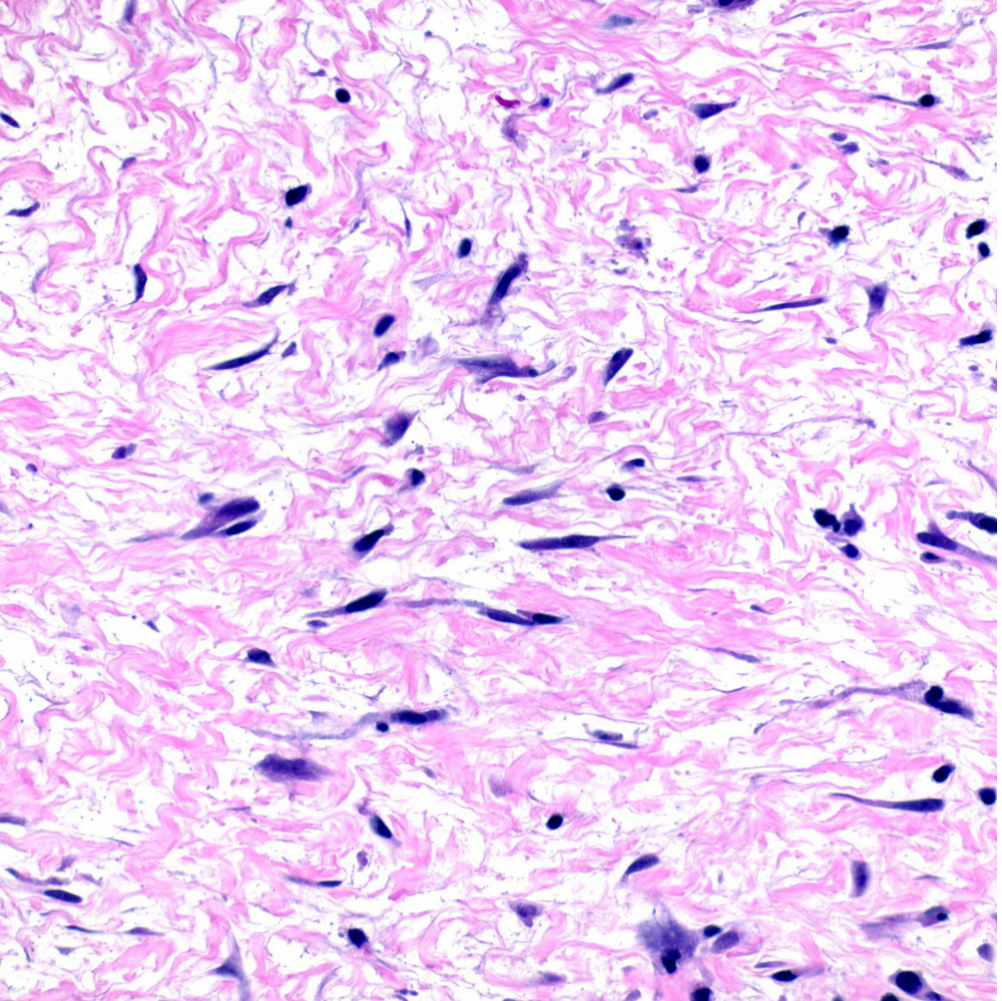
Spindled cells admixed with dermal collagen in a cutaneous lesion of a patient with established nephrogenic systemic fibrosis (H&E stain).

**Figure 2:**
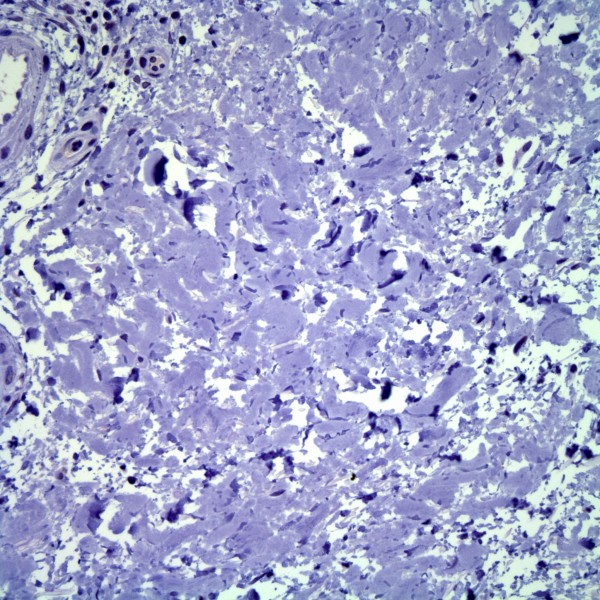
Spindled cells in nephrogenic systemic fibrosis are negative for HHV8 (LNA-1 immunohistochemical stain).

**Figure 3:**
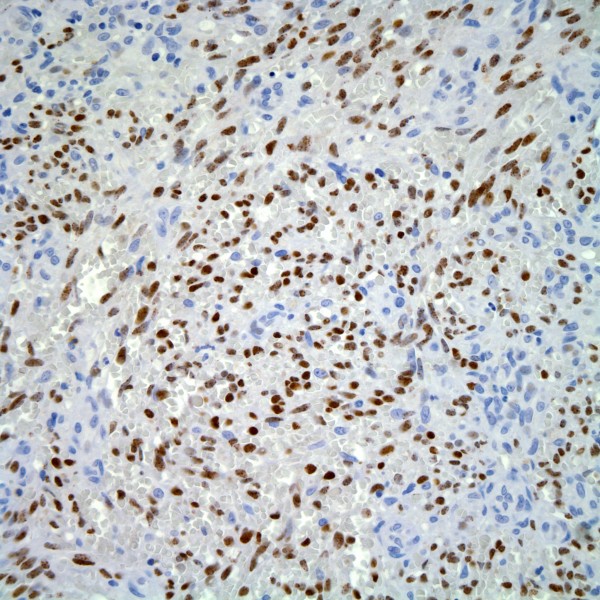
Spindled tumor cells of Kaposi sarcoma (positive control) are strongly immunoreactive for the HHV8 marker LNA-1 (LNA-1 immunohistochemical stain).

We studied tissue from deep punch biopsies of two male NSF patients (73 and 78 years of age), both of whom had a history of chronic renal insufficiency, repeat exposures to gadolinium containing contrast media (Table 1), and recent onset of symmetrical plaques of the distal lower extremities. Certain gadolinium-containing contrast agents, each with a unique chelator molecule non-covalently bound to a Gd3+ ion, are more likely than others to induce NSF [20]. Unfortunately, we were unable to determine the exact dose and type of agent used in these patients, nor the exact time interval between their exposure and onset of NSF. The histologic findings in both individuals were typical of NSF (Figure 1). IHC using a monoclonal antibody to HHV8 (LNA-1, 1:80 dilution, NovoCastra) was negative in both cases (Figure 2), with appropriate positive HHV8 staining in Kaposi sarcoma control cases (Figure 3).

**Table 1:** Renal function in relation to gadolinium exposure (creatinine laboratory reference range = 0.7 – 1.2 mg/dL; BUN laboratory reference range = 8 – 23 mg/dL).

Patient	73-year-old male		78-year-old male	
Gadolinium exposure	Lumbar MRI		Upper extremity MRA	

Renal function	Creatinine mg/dL	BUN mg/dL	Creatinine mg/dL	BUN mg/dL
Before exposure	0.7	21	2.6	85
1^st ^gadolinium exposure	2.3	53	6.0	44
2^nd ^gadolinium exposure	2.4	127	4.7	58
Current (post exposure)	3.1	80	8.6	84

MRI = magnetic resonance imaging. MRA = magnetic resonance angiography. BUN = blood urea nitrogen

## Conclusion

In contrast to KS, we provide evidence that HHV8 appears unlikely to play an etiologic role in the development of NSF. However, this finding is limited to only two patients. Positive immunostaining for HHV8 using LNA-1 exhibits high sensitivity and specificity (close to 100%) for the diagnosis of HHV8-infected tissue, such as Kaposi sarcoma [21,22]. Several studies have also shown that an absence of LNA-1 immunostaining corresponds well with an absence of HHV8 DNA sequences in tissue using polymerase chain reaction [23]. Finally, although the precise pathogenesis remains undetermined, this study does lend further support to the link between NSF and gadolinium exposure in patients with underlying renal disease.
